# Stimulants and Narcotics, Their Mutual Relations; with Special Researches on the Action of Alcohol, Æther, and Chloroform on the Vital Organism

**Published:** 1865-12

**Authors:** 


For sale by S. C. GRIGGS & Co., Lake St., Chicago, Ill.

---

STIMULANTS AND NARCOTICS, THEIR MUTUAL RELATIONS; WITH SPECIAL RESEARCHES ON THE ACTION OF ALCOHOL, ÆTHER, AND CHLOROFORM ON THE VITAL ORGANISM. By FRANCIS E. ANSTIE, M.D., M.R.C.P., Assistant-Physician to Westminster Hospital, Lecturer on Materia Medica and Therapeutics to the School, and formerly Lecturer on Toxicology. Philadelphia: LINDSAY & BLAKISTON. 1865.

This, like the preceding, is a neatly published volume, containing 414 pages. The topics discussed by the author are of the highest degree of interest and importance. But we have, at present, only time and space sufficient to announce the work. It will be noticed more fully at a future time. Meanwhile, it is for sale by S. C. GRIGGS & Co, Lake St., Chicago, Ill.

